# Patients with acute poisoning presenting to an urban emergency department of a tertiary hospital in Tanzania

**DOI:** 10.1186/s13104-017-2807-2

**Published:** 2017-09-16

**Authors:** Ghaniya S. Mbarouk, Hendry R. Sawe, Juma A. Mfinanga, John Stein, Shankar Levin, Victor Mwafongo, Michael S. Runyon, Teri A. Reynolds, Kent R. Olson

**Affiliations:** 1 Emergency Medicine Department, Muhimbili University of Health and Allied Sciences, Dar es Salaam, P.O. Box 54235, Tanzania; 2 Emergency Medicine Department, Muhimbili National Hospital, Dar es Salaam, Tanzania; 3 Department of Emergency Medicine, University of California San Francisco, San Francisco, CA USA; 4 Department of Emergency Medicine, Carolinas Medical Center, Charlotte, NC USA; 5 Emergency and Trauma Care Lead, World Health Organisation, Geneva, Switzerland; 6 California Poison Control System, University of California, San Francisco, CA USA

**Keywords:** Toxicology, Poisoning in Africa, Poisoning diagnosis, ED in Africa

## Abstract

**Background:**

Poisoning is a major public health concern in sub-Saharan Africa, affecting patients of all age groups. Poisoned patients often present to the emergency department (ED) and prompt evaluation and appropriate management are imperative to ensure optimal outcomes. Unfortunately, little is known about the specific presentations of poisoned patients in East Africa. We describe the clinical and epidemiological features of patients presenting to the Muhimbili National Hospital (MNH) ED with suspected toxicological syndromes.

**Methods:**

This prospective study enrolled a consecutive sample of ED patients who presented with a suspected toxicological syndrome from March 2013 to June 2013. Trained investigators completed a structured case report form (CRF) for each eligible patient, documenting the suspected poison, demographic information, the clinical presentation, and the ED outcome and disposition. The study data were analyzed and summarized with descriptive statistics.

**Results:**

Of 8827 patients, who presented to ED-MNH, 106 (1.2%) met inclusion criteria, and all were enrolled. Among those enrolled, the median age was 28 years (interquartile range [IQR] 16 years), and 81 (76.4%) were male. Overall 55 (52%) were single, and 28 (26.4%) had professional jobs. 60 (56.6%) patients were referred from district hospitals, 86.8% of which were in Dar es Salaam. Only 13 (12.3%) of patients presented to the ED within 2 h of the toxic exposure. The etiology of poisoning included alcohol in 42 (50%), a mixture of different medications in 12 (14.3%), and snakebite in 6 (11.3%). Most exposures were intentional (63 [59.4%]) and were via the oral route (88 [83%]). The most common abnormal physical findings were altered mental status (66 [62.3%]) and tachypnoea (68 [64.2%]). One patient died in the ED and 98 (92.5%) required hospital admission.

**Conclusions:**

Most patients presenting to the ED with a toxicological syndrome were adult males with intentional exposures. The most common toxic exposure was alcohol (ethanol) intoxication and the most common abnormal findings were altered mental status and tachypnoea. More than three-quarter of patients presented after 2 h of exposure. Almost all patients were admitted to the hospital.

## Background

Acute poisoning is a major medical emergency that carries significant morbidity and mortality in patients of all age groups across the world [1–4]. The World Health Organization (WHO) estimates that poisoning results in an annual loss of 7.4 million years of healthy life (disability adjusted life years) globally. However, this burden disproportionally impacts low and middle-income countries, where over ninety percent of deaths due to unintentional poisoning occur [5].

Acute poisoning may be intentional or unintentional, and studies have shown that intentional poisoning is more common in adults and accidental poisoning is more common in children [6]. Patients with acute poisoning have highly variable clinical presentations, making diagnosis difficult, especially in resource limited settings [7, 8]. The variability of the clinical presentation is further compounded by the fact that substances involved in poisonings vary by age group, intention, geographic region, and level of economic development [6, 9–11]. In the United States, the American Association of Poison Control Centers reports that the top five substance classes most frequently involved in human exposures are analgesics, cosmetics, household cleaning substances, sedatives/hypnotics/antipsychotics, and foreign bodies [8]. European data indicates a different pattern, reflective of the fact that most poisoned patients are adults with suicidal intention, with illicit drugs (mostly benzodiazepines), sedatives/hypnotics/antipsychotics, alcohol, and carbon monoxide as the most common exposures [10].

In Sub Saharan Africa, there is a general paucity of Emergency Department (ED) data describing patterns of acute poisoning, with only a few hospital and mortuary based studies [12–14]. In Tanzania, ED based studies reporting on acute poisoning are non-existent, but poisonings have been documented in other clinical settings. In one study of suicides conducted in the mortuary of the main tertiary referral hospital, poisoning was the most common method suicide, mainly from ingesting antimalarials and pesticides [15]. Another autopsy based surveillance study conducted in southern Tanzania documented 300 accidental cyanide poisoning deaths from consumption of bitter cassava during a country-wide drought in 2002–2003 [16]. Poisonings, have also been documented in mining operations (occupational exposures) and among affected pregnant women presenting to a tertiary referral hospital [17–19].

In acute settings, early recognition and appropriate management have been known to improve outcomes of poisoned patients [20]. Delayed recognition and sub-optimal management may lead to increased morbidity and mortality [21]. The case fatality rates vary by poison and region. In Africa and Asia, fatalities have been shown to be highest from pesticides, followed by medications and household products, while paracetamol was found to be a significant cause of poisoning in other regions of the developing world [6].

In high resource settings, poison control centres are common [22] and most emergency physicians managing poisoned patients have the benefit of real time consultation with the expert toxicologists staffing those centres. Thus, in most EDs in the developed world, physicians are able quickly identify the type of poisoning and institute appropriate management, increasing the likelihood of satisfactory outcomes [23]. Unfortunately, there are currently no formal Poison Control Centres or toxicological consultation services readily available to providers in most Sub Saharan Africa countries, including Tanzania. Recognizing toxic cases is still a challenge in Tanzania because there are relatively few acute care providers with the training necessary to detect the subtle and variable presentations of toxicological syndromes. This issue is compounded by lack of access to laboratory toxicological analysis.

Therefore, we aimed to describe the population of patients presenting to the ED of a large public tertiary care hospital with suspected acute poisoning or other toxic exposure, characterizing their presenting signs and symptoms, and reporting on suspected toxicological agents involved. This was hoped to increase providers’ knowledge of patients with suspected toxicological syndromes presenting to EDs and other acute intake areas in Tanzania, thereby informing the development of diagnostic and management protocols and guiding educational and clinical initiatives to reduce the morbidity and mortality from toxicological disease.

## Methods

### Study design

This was a prospective observational study of a consecutive sample of patients presenting to the MNH-ED from 15th March 2013 to 17th June 2013.

### Study setting and population

MNH is a tertiary referral hospital in Dar es Salaam, Tanzania and the main clinical training site for the Muhimbili University of Health and Allied Sciences (MUHAS). The ED was opened in 2010, and it is the site for the only emergency medicine residency program in the country. The MNH-ED receives high acuity patients from within Dar es Salaam and the surrounding regional and district hospitals and served 50,000 patients in 2013. The top five patient presentations are trauma, infectious disease, mental health, neoplasm and pregnancy related complications [24].

The department is staffed by locally trained emergency physicians, who provide clinical care, supervision and teaching to interns (fresh graduates from medical school), registrars (generalists) and emergency medicine residents.

In this study, all patients presenting with suspected poisoning were eligible for enrollment. Suspicion of poisoning was determined by the treating physician based on the history, suggestive clinical picture or presence of the containers or wrappers of the poisonous agent or medications.

### Study protocol

#### Operational definition

Toxicological syndrome was defined as a group of signs and symptoms constituting the basis for a diagnosis of poisoning.

#### Inclusion criteria

All patients presenting to MNH ED with provider impressions including but not limited to: alcohol intoxication, recreational or prescription drug overdose, toxic inhalation, chemical skin exposure, accidental or intentional poisoning, and those who had a snakebite or envenomation.

The treating physicians screened and enrolled patients consecutively with support monitoring by one of the study authors (GM). Data collection was done 24 h everyday and 7 days per week. Providers obtained informed consent from all eligible patients or their accompanying eligible proxy. A standardized case report form (CRF) with explicitly defined variables was used to prospectively collect the study data, including age, sex, occupation and other demographic data, as well as substance(s) suspected, details of exposure, prior psychiatric history, household medications, clinical presentation, ED outcomes and disposition.

### Data analysis

The study data recorded in the CRF were transferred into an Excel database (Microsoft corporation, Redmond, WA), cleaned, and analyzed with Excel and Stata (version 13, StataCorp LP, Texas, USA). Continuous variables were summarized as means and standard deviations (SD) or medians and interquartile ranges (IQR), depending upon the data distribution. Categorical variables are summarized as counts and percentages.

## Results

### Study population demographics and referral status

Out of 8827 patients who presented to ED-MNH, 106 (1.2%) met inclusion criteria and all were enrolled. The majority (81 [76.4%]) were male and over the age of 18 years, (84 [79.2%]). The overall median age was 28 years (interquartile range [IQR] 16 years; range 1 month to 91 years) (Table 1). Most patients were single, and nearly one-third had a primary level of education or less.

**Table 1:** Demographic characteristics of the patients

Age and gender	N = 106
Age	
Median (IQR)	28 (16) years
Age groups (years)	
<5	11 (10.4%)
5 to <18	11 (10.4%)
≥18	84 (79.2%)
Gender	
Male	81 (76.4%)
Female	25 (23.6%)
Marital status	
Single	55 (51.9%)
Married	35 (33.0%)
Others^a^	3 (2.8%)
Unknown	13 (12.3%)
Level of education	
Primary school	34 (32.1%)
Secondary school	25 (23.6%)
College and University	17 (16.0%)
None	13 (12.3%)
Unknown	17 (16.0%)
Occupation	
Professional job^b^	28 (26.4%)
Unemployed	18 (17%)
Student	17 (16%)
Others^c^	11 (10.4%)
Driver	10 (9.4%)
Guard	10 (9.4%)
Farmer	2 (1.9%)
Unknown	10 (9.4%)

^a^Includes divorced, widowed

^b^Nurse, Engineer, Lawyer, Businessman, Secret service, Machine operator, Secretary, Surveyor, Veterinary doctor, Social worker, Accountant

^c^Barman, cook, builder, porter, housewife, shoe shiner, gardener, miner, bus assistant and maid

### Geographical location of exposure, referral status and time of arrival

Most toxic exposures occurred within the Dar es Salaam region (92 [86.8%]), with the highest rate in the Kinondoni district (35 [38.0%]) compared to the Ilala (29 [31.5%]) and Temeke (18 [19.6%]) districts (all of which are within Dar es Salaam), Most exposures occurred at home [41 (38.7%)] or recreational places (36 [34.0%]) (Table 2).

**Table 2:** Geographical location of exposure, referral status and time of arrival

Region	
Dar es Salaam	92 (86.8%)
Coastal	9 (8.5%)
Other regions^a^	5 (4.7%)
Place	
Home	41 (38.7%)
Recreational area	36 (34.0%)
At work	10 (9.4%)
Farm	1 (0.9%)
Others^b^	18 (17.0%)
Referral status	
District hospital	60 (56.6%)
Self referral	26 (24.5%)
Brought by police	13 (12.3%)
Others^c^	7 (6.6%)
Presenting time after exposure	
<2 h	13 (12.3%)
2–12 h	42 (39.6%)
>12 h	32 (30.2%)
Unknown exposure time	19 (17.9%)

^a^Includes Kigoma, Mtwara, Dodoma and unknown region

^b^Includes on the street, school, mosque, hostel and unknown

^c^Includes referral from private hospitals, regional hospital and MNH methadone clinic

Just over one half of patients were referred from district hospitals, and one quarter presented directly to the MNH-EMD.

Only 13 (12.3%) presented to the MNH-EMD within 2 h after the toxic exposure. The largest group (42 [39.6%]) presented within 2–12 h followed by (32 [30.2%]) who presented after more than 12 h (Table 2).

### Patients’ clinical presentation and laboratory result

Most patients had no psychiatry history (90 [84.9%]) and were not on chronic medications (92 [86.8%]). Most patients presented with normal temperatures (101 [95.3%]), normal random blood glucose (92 [86.8%]), normal-sized pupils (83 [78.3%]), normal skin findings (78 [73.6%]) and normal pulse rate (69 [65.1%]). The most common abnormal findings included tachypnoea (68 [64.2%]) and altered mental status (66 [62.3%]) (Table 3). A small number of patients 7 (6.6%) had a mixture of symptoms, including abdominal cramps, swollen lower limb, excessive salivation, convulsions, urinary retention, bloody secretions, neck and jaw stiffness and lethargy.

**Table 3:** Patients’ clinical presentation and laboratory results

Characteristic	Findings		
Vital signs	Normal	Low	High
Temperature (N = 106)	101 (95.3%)	0	5 (4.7%)
Pulse rate (N = 106)	69 (65.1%)	5 (4.7%)	32 (30.2%)
Respiratory rate (N = 106)	36 (34.0%)	2 (1.9%)	68 (64.2%)
MAP (N = 93)^a^	43 (46.2%)	4 (4.3%)	46 (49.5%)
Clinical signs			
Pupil character	Normal	Miotic	Mydriatic
N = 106	83 (78.3)	17 (16.0%)	5 (4.7%)
Skin status	Normal	Dry	Sweaty
N = 106	78 (73.6%)	15 (14.2%)	13 (12.3%)
Mental status	Normal	Altered	Agitated
N = 106	40 (37.7%)	66 (62.3%)	14 (13.2%)
Gastrointestinal	Normal	Vomiting	Diarrhoea
N = 106	84 (79.2%)	14 (13.2%)	8 (7.6%)
Genital urinary	Normal	Urine incontinence	
N = 106	95 (89.6%)	11 (10.4%)	
Laboratory results			
Random blood glucose	Normal	Low	High
N = 106	92 (86.8%)	6 (5.7%)	8 (7.5%)

^a^Mean arterial pressure

### Suspected agents for all age groups

The top two toxic agents encountered by adult patient were alcohol (42 [50%]) taken alone, and mixtures of different medications (12 [14.3%]), including alcohol plus sedatives, ethanol plus rat poisoning, ethanol plus antimalarial and antibiotics, ethanol plus unknown agents. No specific agent was identified in 8 (9.5%) of adult patients.

In children below the age of 18 years top three toxic agents were snake venom (6 [27.3%]), kerosene (3 [13.6%]) and rat poison (3 [13.6%]) (Table 4).

**Table 4:** Suspected agents in all patients

Adult ≥18 years	
Toxic agent	N = 84
Alcohol	42 (50.0%)
Mixture of different medications^a^	12 (14.3%)
Unknown	8 (9.5%)
Sedative or hypnotic agent	7 (8.3%)
Organophosphates	7 (8.3%)
Snake venom	6 (7.1%)
Rodenticide (rat poison)	5 (5.9%)
Anti-malarial (Artemisinin based combination)	2 (2.4%)
Tricyclic antidepressants	2 (2.4%)
Opioids	2 (2.4%)

Children (<18 years)	
Toxic agent	N = 22
Snake venom	6 (27.3%)
Kerosene	3 (13.6%)
Rat poison	3 (13.6%)
Poly pharmacy	2 (9.1%)
Insecticide	1 (4.6%)
Acid	1 (4.6%)
Antimalarial	1 (4.6%)
Pesticide	1 (4.6%)
Tricyclic antidepressant	1 (4.6%)
Organophosphate	1 (4.6%)

^a^Includes: ethanol plus sedatives, ethanol plus rat poisoning, ethanol plus antimalarial and antibiotics, ethanol plus unknown agents

### Route of exposure and intent for all age groups

Overall, nearly 63 (59.4%) of the toxic exposures were intentional, and intentional poisoning was most common in adults 59 (95.2%). Most common route of exposure was oral (88 [83.0%]), followed by bites or stings by venomous animal/insects (11 [10.4%]) (Table 5).

**Table 5:** Route of exposure and intent for all age groups

Route of exposure	N **=** 106			
Oral-ingestion	88 (83.0%)			
Bite or sting by venomous animal/insects	11 (10.4%)			
Skin	5 (4.7%)			
Inhaled	3 (2.8%)			
Unknown	3 (2.8%)			

Intent				
Intentional exposure	Overall N = 106	Recreational N = 63	Suicidal N = 63	Assault N = 63
Frequency (%)	63 (59.4%)	37 (34.9%)	16 (15.1%)	10 (9.4%)

Unintentional exposure	Overall N = 106	Accidental N = 37	Drug reaction N = 37	Occupational N = 37
Frequency (%)	37 (34.9%)	35 (33%)	2 (1.9%)	0
Unknown	6 (5.7%)			

### Patient disposition and outcome

Overall, most of the patients (98 [92.5%]) were admitted to the hospital, 7 (6.6%) were discharged home from the ED, and one patient (0.9%) died in the ED.

## Discussion

Most of the patients in our study population were from Dar es Salaam city, reflecting similar patterns of referral to the MNH ED shown in previous studies [25]. We found that the majority of acute poisoning among patients presenting to our ED in Tanzania are males, with a 3:1 male to female ratio. These findings are similar to other studies done in Sub Saharan Africa, where acute poisoning is known to be common among young males [26]. In our study population, single, adult males with a limited level of education represented nearly one-third, which is consistent with a study by Ndosi et al. on fatal self-poisoning patients in Tanzania in which most of the patients were single, with a primary level of education [15]. However, contrary to observations made in other studies in similar settings [15, 26], over one-quarter of our patients had professional jobs (including nurse, engineer, lawyer, businessman, and accountant), with most of these professionals presenting with intentional, recreational poisonings.

Contrary to observations made in most high-income countries [27, 28], alcohol taken for recreational purposes was found to be the most common toxic agent in our study population. These findings are different from earlier studies of poisoning done in Tanzania which showed that the most common substances used for fatal self-poisoning were antimalarials and pesticides. This difference may be due to our inclusion of all patients with suspected toxic exposure regardless of intentionality or suicidality, while both previous studies evaluated deceased patients or those with suicidal intention.

In evaluating children as a subset of our study population, we found that most common toxic exposure was snakebite, followed by accidental ingestion of kerosene or rodenticides. Contrary to findings of previous studies in similar settings [26] our findings of snakebites are interesting and suggests the need for a larger multicenter study which can lead to dedicated protocols of snakebite management. We were unable to identify the exact contents of the rodenticides but the most common products available in Tanzania include warfarins and related anticoagulants, zinc or aluminium phosphide, and organophophates. Despite having no reported deaths in the group of children who had rat poisoning, the diversity of the contents and serious potential toxicity continue to pose a significant challenge.

Nearly two-thirds of the toxic exposures occurred at home or in recreational areas, and over half of the toxic exposures were intentional. Intentional poisoning was five times more common in adults than in children. Most children had their toxic exposure at home, similar to other studies from sub-Saharan Africa [29], which have suggested that the culture of leaving children unattended is one important risk factor for domestic based accidents, including poisoning [29, 30]. Most of the intentional toxic exposures were due to recreational use of alcohol and only about a quarter were due to suicidal intention. Community based behavioral interventions [31] may be part of the solution to tackling acute alcohol poisoning incidents in Tanzania.

Clinical presentations of toxic exposures depend on the specific agent involved, the quantity absorbed, and the intent of exposure. The most common presenting clinical feature in our study population was altered mental status, which occurred in over 60% of patients. The differential diagnoses and management strategies of patients with altered mental status are widely variable [32], and health providers should always consider poisoning as a potential cause for altered mental status in patients presenting to acute intake areas in Tanzania.

Conservative management-including intravenous fluids and anti-pain-was the main stay of treatment for our patients, and over 90% of these patients were admitted to the hospital, three of who were intubated and admitted to intensive care units (ICU). In our setting mechanical ventilation and ICU care is significantly limited by availability of acute care beds and ventilator machines [33]. As such, we believe it is inappropriate to use ICU admission or intubation as markers of illness severity in this setting. Less than 10% of patients were discharged from the ED, half of who had a discharge diagnosis of alcohol intoxication. All patients who were discharged had stable vital signs; however, due to the nature and timing of the study, and the available resources, we were unable to follow up the discharged patients to investigate their outcomes after discharge. In this study, one patient died in the ED and the toxic substance that was involved was unknown and, as is common in our setting, no postmortem examination was performed.

## Limitations

This was a single site descriptive study with a small sample size over a short period of time, at the ED of a large public tertiary referral centre, hence our results may not necessarily be generalizable to other EDs with different patient populations in difference settings. Furthermore, the potential for seasonal variations might not be ascertained within our findings. We relied on the treating doctors’ clinical impression to identify eligible patients. These doctors may have variable ability to diagnose toxicological syndromes depending on their level of training and experience, and we have no laboratory analysis capabilities for toxicology screening. As such, we suspect that many toxicology patients pass through the MNH-EMD undiagnosed.

## Conclusions

The majority of patients presenting to our ED with poisoning are adult male with intentional exposures, mostly after recreational use of alcohol. Substances used in adult suicidal ingestions included rodenticides, acid, antimalarials, organophosphates, tricyclic antidepressants, haloperidol and opioids. Substances involved in childhood poisonings included kerosene, rodenticides, antimalarials, antibiotics and ethanol. Future studies should focus on larger multicentre patient population and laboratory confirmation of the suspected poisoning agents.

## Abbreviations

ED: emergency department
CRF: case report form
WHO: World Health Organization
MNH: Muhimbili National Hospital
CBC: complete blood count
MUHAS: Muhimbili University of Health and Allied Sciences
